# Creating space for Indigenous healing practices in patient care plans

**DOI:** 10.36834/cmej.68647

**Published:** 2020-03-16

**Authors:** Lindsey Logan, Jacinta McNairn, Shelley Wiart, Lynden Crowshoe, Rita Henderson, Cheryl Barnabe

**Affiliations:** 1 Cumming School of Medicine, University of Calgary, Alberta, Canada; 2 Athabasca University, Alberta, Canada

## Abstract

**Background:**

The Truth and Reconciliation Commission of Canada’s Calls to Action ask that those who can effect change within the Canadian healthcare system recognize the value of Indigenous healing practices and support them in the treatment of Indigenous patients.

**Methods:**

We distributed a survey to the Canadian Rheumatology Association membership to assess awareness of Indigenous healing practices, and attitudes informing their acceptance in patient care plans.

**Results:**

We received responses from 77/514 members (15%), with most (73%) being unclear or unaware of what Indigenous healing practices were. Nearly all (93%) expressed interest in the concept of creating space for Indigenous healing practices in rheumatology care plans. The majority of support was for the use in preventive or symptom management strategies, and less as adjuncts to disease activity control. Themes identified through qualitative analysis of free-text responses included a desire for patient-centered care and support for reconciliation in medicine, but with a colonial construct of medicine, demonstration of an evidence bias, and hierarchy of medicines.

**Conclusions:**

Overall, respondents were open to the idea of inclusion of Indigenous healing practices in patient’s car plans, emphasizing importance for patient empowerment and patient-centered care. However, they cited concerns that provide the indication for further learning and reconciliation in medicine.

## Abstract (fr)

**Contexte:**

Les appels à l’action de la Commission de vérité et réconciliation du Canada demandent à ceux qui peuvent effectuer des changements au sein du système de soins de santé canadien de reconnaître la valeur des pratiques de guérison autochtones et de les soutenir dans le traitement des patients autochtones.

**Méthodes:**

Nous avons distribué un sondage aux membres de la Société canadienne de rhumatologie pour évaluer leurs connaissances des pratiques de guérison autochtones et leurs positions quant à l’acceptation de ces pratiques dans les plans de soins des patients.

**Résultats:**

Nous avons reçu des réponses de 77 membres sur 514 (15 %). La plupart (73 %) étaient incertains ou pas au courant de ce que sont les pratiques de guérison autochtones. Presque tous (93 %) ont exprimé un intérêt dans le concept de création d’un espace pour les pratiques de guérison autochtones dans les plans de soins en rhumatologie. La plus grande partie du soutien avait trait davantage à leur utilisation dans des stratégies de prévention ou de gestion des symptômes, et moins comme des auxiliaires dans le contrôle de l’activité de la maladie. Les thèmes relevés par une analyse qualitative des réponses en texte libre comprenaient un désir pour des soins centréssur le patient et un soutien pour une réconciliation en médecine, mais avec un concept colonial de médecine, une démonstration de l’existence d’un biais et une hiérarchie des médicaments.

**Conclusions:**

Dans l’ensemble, les répondants demeuraient ouverts à l’idée d’inclure des pratiques de guérison autochtones dans les plans de soinsdes patients, mettant l’accent sur l’importance de la capacité d’agirdu patient et des soins centréssur le patient. Toutefois, ils mentionnaient des préoccupations qui donnent une indication pour un plus grand besoin d’apprentissage et de réconciliation en médecine.

## Introduction

Inequities in Indigenous Peoples’ health status heighened through policies and legislation aimed at controlling and colonizing Indigenous populations such as the residential school system,^1^ while removing access to traditional wellness practices.^2^ Inequities are perpetuated to this day through pervasive health effects of these events such as intergenerational trauma, and through current sociopolitical organization. While the dismantling of structural barriers is ultimately required, particular interventions support improved health outcomes. Receiving culturally appropriate care free of racism and stereotyping, that supports relationship building and promotes engagement of culture to support disease management, has contributed to enhanced diabetes care.^3^^,^^4^ Reintroducing Indigenous healing practices improved ownership over choices around, and access to, diverse health systems.^5^ Further, cultural continuity, implying utilization of healing practices, guards against the development of diabetes^6^ and the occurrence of suicide.^7^ However, these approaches have not been broadly realized in western health systems that privilege biomedical approaches and western knowledge paradigms and models.^8^

Global organizations, including the World Health Organization (WHO) and the United Nations, support the availability of traditional health practices. The WHO provides a definition of traditional medicine, including Indigenous healing practices, which is: “*The sum total of the knowledge, skill, and practices based on the theories, beliefs, and experiences indigenous to different cultures, whether explicable or not, used in the maintenance of health as well as in the prevention, diagnosis, improvement or treatment of physical and mental illness*”.^9^ The rights to traditional medicines and health practices are declared in Article 24 of the United Nations Declaration on the Rights of Indigenous Peoples (UNDRIP).^10^ Further, the Truth and Reconciliation Commission (TRC) of Canada’s 94 Calls to Action (2015) included the following, Call to Action #22: ‘*We call upon those who can affect change within the Canadian health-care system to recognize the value of Aboriginal healing practices and use them in the treatment of Aboriginal patients in collaboration with Aboriginal healers and Elders where requested by Aboriginal patients’*.^11^

In light of the Canadian government’s acceptance of UNDRIP, the TRC Calls to Action, and the medical community’s evolving emphasis on patient-centered care, there is impetus for medical practitioners to support patients’ decisions to use Indigenous healing practices. This is particularly relevant in rheumatology. Indigenous patients in Canada experience a prevalence of arthritis that exceeds the general population rates^12^ with inequitable disease outcomes.^13^ The discipline’s focus is on abrogation of inflammation and symptom management, often requiring long-term treatment and mitigation plans. We thus conducted a survey to explore rheumatologists’ awareness of Indigenous healing practices and their views on the inclusion of these practices in patient care plans, encompassing herbal medicines, Indigenous wellness counselling, and spiritual/ceremonial practices. The purpose of the work was not to validate Indigenous healing practices within the medical community, but rather to explore physicians’ perceptions, and identify barriers to support this aspect of patient-centered care for Indigenous patients.

## Methods

### Study design

The research team developed a survey based on a prior study of Canadian family physicians’ attitudes toward patients’ use of ‘traditional native medicines.’^14^ We engaged in a collaboration with the Canadian Rheumatology Association, thus the survey was tailored to modern rheumatology practice. The survey included general demographic questions and questions about the respondents’ awareness/understanding of Indigenous healing practices. Within the survey, we provided the WHO definition of traditional medicine^9^ in reference to Indigenous healing practices. We inquired about respondents’ perceptions on the inclusion of particular Indigenous healing practices: herbal medicines (therapeutic use of plant materials), Indigenous wellness counselling (use of relationships in healing), and spiritual/ceremonial practices (activities founded on traditional knowledge and spiritual belief). In the survey, examples of specific Indigenous healing practices were not provided, as these knowledges are not appropriately shared in this type of format, and to as not bias the respondents in their responses. We asked about their opinions on the inclusion of these healing practices in various contexts, such as symptom management (management of pain, stiffness, swelling), health maintenance and preventive care, and disease management (treatment of disease activity). Our cross-sectional survey included multiple choice questions, Likert scales, and free-text fields. Questions related to the physician’s comfort in inquiring about the use of Indigenous healing practices, perceived health risks of these practices, and preferred learning formats requested further explanation, elaboration and suggestions by the respondent, respectively. Additional comments were requested at the end of the survey. A recruitment email with a link to the survey in English and French (Appendix A) was distributed by the Canadian Rheumatology Association to the membership (n=514) in the fall of 2018. Responses were collected using the Survey Gizmo platform. Survey responses were accepted over a 4-week period with a reminder email sent at 2 weeks.

### Research team description

The research team included two medical students (LL and JM), who approached two Indigenous academic physicians (LC – family medicine and CB - rheumatologist) with the desire to support advancing Indigenous Peoples’ health. Together these four investigators developed the quantitative survey and completed response analysis. Qualitative analysis was completed by the two medical students along with an Indigenous graduate student (SW) and a settler medical anthropologist who holds an academic appointment as a models of care scientist and who has been deeply engaged in Indigenous health research (RH). All research team members considered the combined quantitative and qualitative data results in the interpretation of the study and assembling the manuscript.

### Ethics

The study was approved by the University of Calgary Conjoint Health Research Ethics Board (REB 18-1081) and distributed to the CRA Membership after approval of the Communications Committee and the organization’s CEO and Board.

### Data analysis

Quantitative Analysis: Descriptive statistics (mean, standard distribution, frequency) were used to summarize quantitative data responses. Chi-squared testing was used to identify whether Likert-scale responses varied with participant demographics, practice demographics, or participation in clinical service outreach to Indigenous communities. Analysis was completed using Stata (College Station, Texas; version 11.2).

Qualitative Analysis: A nominal group technique^15^ was used to promote a consensus-based, non-hierarchical collaborative process for free-text response analysis. Three team members (JM, LL, SW) worked independently to identify major themes reflected in responses. They then met as a group, shared and discussed each theme, and unanimously agreed to a common coding framework. They then worked independently to code each free-text response to one or more of the revised themes. The team met a second time to establish consensus regarding the final theme wording and response coding.

## Results

### Respondent demographics

We distributed the survey to 514 CRA members, and received 77 responses (15%). Respondents (none of whom were Indigenous) ranged across age cohorts and provinces/regions, with the majority having trained between 1976 and 2009 in Canadian centres. Most were providing care to adult patients. One quarter of respondents had greater than 10% of Indigenous patients in their practice with 16% of respondents providing outreach care to Indigenous communities (Table 1).

**Table 1:** Demographic characteristics of survey respondents

Demographic Characteristic		N (%) n=77
Age	<40 years	30 (39%)
	41-60 years	30 (39%)
	> 60 years	17 (22%)
Year of Graduation	Prior to 1975	6 (8%)
	1976 to 2009	50 (65%)
	After 2009	21 (27%)
Country of Medical School Graduation	Canada	63 (82%)
	Other	14 (18%)
Primary Practice Location	British Columbia	16 (22%)
	Alberta	14 (19%)
	Saskatchewan/Manitoba	8 (11%)
	Manitoba	5 (7%)
	Ontario	22 (30%)
	Quebec/Atlantic Canada	14 (19%)
	Territories	0 (0%)
Primary Practice Setting	Urban	76 (100%)
Primary Practice Type	Paediatric	13 (17%)
	Adult	63 (82%)
Outreach to Indigenous Communities	Yes	12 (16%)
Proportion of Patients who are Indigenous	<10%	57 (74%)
	11-50%	19 (25%)
	>50%	1 (1%)

### Awareness

**Figure 1:**
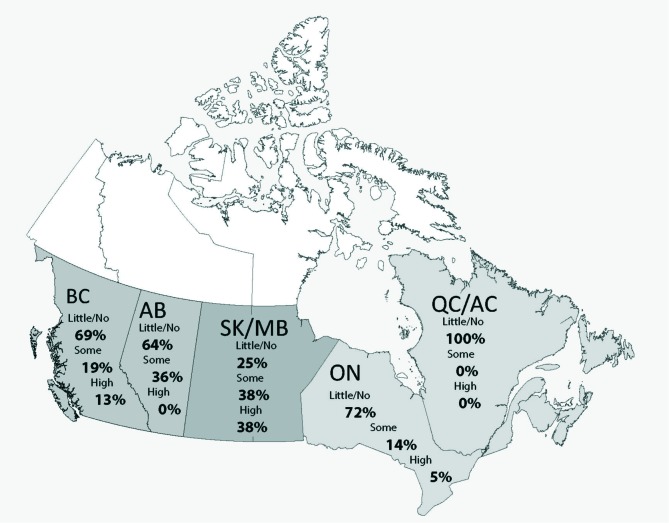
Self awareness rating distribution by primary province of practice.

Most respondents (73%) rated themselves as unaware of Indigenous healing practices, and 40% reported no previous exposure to Indigenous healing practices (Table 2). We found an association between self-reported level of awareness and practice location, with the highest level of awareness reported in Saskatchewan and Manitoba, and the lowest level of awareness amongst rheumatologists practising in Ontario, Quebec, and Atlantic Canada (p=0.001, Figure 1). Awareness was also higher in rheumatologists who had themselves participated in Indigenous healing practices, observed their practice, or sought Indigenous healing services for patients (p<0.01). Awareness did not vary by rheumatologist age, year of graduation from medical school, or location of medical training.

**Table 2:** Awareness of Indigenous healing practice

Survey Response	N
**How would you rate your awareness of Indigenous healing practices? (n=77)**	
1 (no previous exposure)	28 (36%)
2	28 (36%)
3 (somewhat aware)	14 (18%)
4	5 (7%)
5 (have actively engaged in learning about Indigenous healing practices)	2 (3%)
**In what ways have you been previously exposed to Indigenous healing practices? (n=75)**	
No previous exposure	30 (40%)
In medical school/residency	20 (27%)
Through reading and/or media	28 (37%)
Have observed its practice	18 (24%)
Have participated in Indigenous healing activities	4 (5%)
Have sought out Indigenous healing for my patients	2 (3%)
**Are you aware of patients in your practice using Indigenous healing practices to treat or supplement treatment of rheumatological condition? (n=77)**	
Yes	38 (49%)
No	39 (51%)
**What forms of Indigenous healing practices are your patients using? (n=37)**	
Herbal	28 (76%)
Ceremonial/spiritual	33 (89%)
Wellness counselling	13 (35%)
Other	2 (5%)
**How comfortable are you inquiring about the use of Indigenous healing practices with patients? (n=77)**	
Extremely uncomfortable	4 (5%)
Uncomfortable	6 (8%)
Neutral	30 (39%)
Comfortable	35 (46%)
Extremely comfortable	2 (3%)
**If a patient were to request an Indigenous healer or elder while in clinic or in hospital, would you be able to find one for them? (n=76)**	
Yes	28 (37%)
No	48 (63%)

Ratings are grouped together as little/no awareness (Likert scale response 1 or 2), some awareness (Likert scale response 3), and high awareness (Likert scale response 4 or 5). We found a significant effect (p=0.001) of province of practice on awareness of Indigenous healing practices. Map template retrieved from FreeVectorMaps.Com (CA-EPS-01-0005 ©).

Most survey respondents were comfortable or neutral inquiring about the use of Indigenous healing practices, and half reported being aware of patients in their practice using these approaches, in various forms (e.g., herbal, spiritual, wellness counselling, Table 2). The only demographic characteristic associated with higher comfort levels was participation in clinical outreach services (p=0.021).

### Indigenous healing practices in patient care plans

Most respondents (93%) reported being open to the idea of including Indigenous healing practices in rheumatology care plans, although those who graduated prior to 1976 were less open (67% vs >95% in other categories, p=0.027). More than half of respondents (58%) felt that there are potential health risks associated with this, with the most frequent perceived risks including the inability to assess risks due to lack of knowledge (77%), potential of healing practices to interfere with prescribed medications (77%), and potential interference with biomedical treatment plans, including patient adherence to western-based therapy (68%).

Respondents reported higher levels of agreement with the use of spiritual/ceremonial practices and wellness counselling than herbal medicines (Table 3). There was an association between the age of respondents and their level of agreement with the use of Indigenous herbal medicines. Respondents 40-60 years old indicated higher agreement with their use for disease management (p=0.033), symptom management (p=0.043) and inpatient chronic illness (p=0.046) than their older (>60) and younger (<40) counterparts. Respondents generally agreed less with the use of Indigenous healing practices for disease management than for the other phases of care (i.e., health maintenance/preventive care, symptom management, and chronic illness)

**Table 3:** Level of agreement with integration of different types of Indigenous healing practices into patient care plans.

	Clinical Setting	Strongly Disagree	Disagree	Neutral	Agree	Strongly Agree
**Herbal Medicines**	Health maintenance and preventive care	1 (1%)	3 (4%)	26 (35%)	37 (50%)	7 (10%)
	Disease management	6 (8%)	14 (19%)	36 (49%)	18 (24%)	0 (0%)
	Symptom management	2 (3%)	6 (8%)	19 (26%)	40 (54%)	7 (10%)
	Chronic illness, inpatient	4 (6%)	7 (10%)	36 (49%)	22 (30%)	4 (6%)
**Spiritual/Ceremonial Practices**	Health maintenance and preventive care	0 (0%)	2 (3%)	11 (15%)	36 (49%)	24 (33%)
	Disease management	3 (4%)	10 (14%)	21 (29%)	25 (34%)	14 (19%)
	Symptom management	0 (0%)	4 (6%)	11 (15%)	36 (49%)	22 (30%)
	Chronic illness, inpatient	2 (3%)	5 (7%)	15 (20%)	30 (41%)	22 (30%)
**Wellness Counselling**	Health maintenance and preventive care	0 (0%)	1 (1%)	11 (15%)	34 (47%)	27 (37%)
	Disease management	2 (3%)	4 (6%)	26 (36%)	23 (32%)	18 (25%)
	Symptom management	0 (0%)	2 (3%)	17 (23%)	30 (41%)	25 (34%)
	Chronic illness, inpatient	1 (1%)	4 (6%)	19 (26%)	24 (33%)	25 (34%)

### Approaches to creating space for Indigenous healing practices in patient care

Respondents supported various approaches for the inclusion of Indigenous healing practices in patient care, including patient liaison between the rheumatologist and an Elder/healer (43%), Elder/healer as consultant to the rheumatologist (23%), or Elder/healer as a formal team member (25%). One fifth supported full recognition, regulation and funding of Indigenous healing practices by provincial health care systems, whereas half supported partial inclusion in public health care programs. When asked about factors that made them hesitant to include Indigenous healing practices in patient care plans, respondents noted insufficient information on how to refer patients to these services (40%), and/or lack of support from their provincial or institutional health care system (40%). If a patient were to request an Indigenous Elder/healer while in clinic or hospital, 37% of respondents said they would be able to facilitate the request.

### Qualitative themes

**Figure 2:**
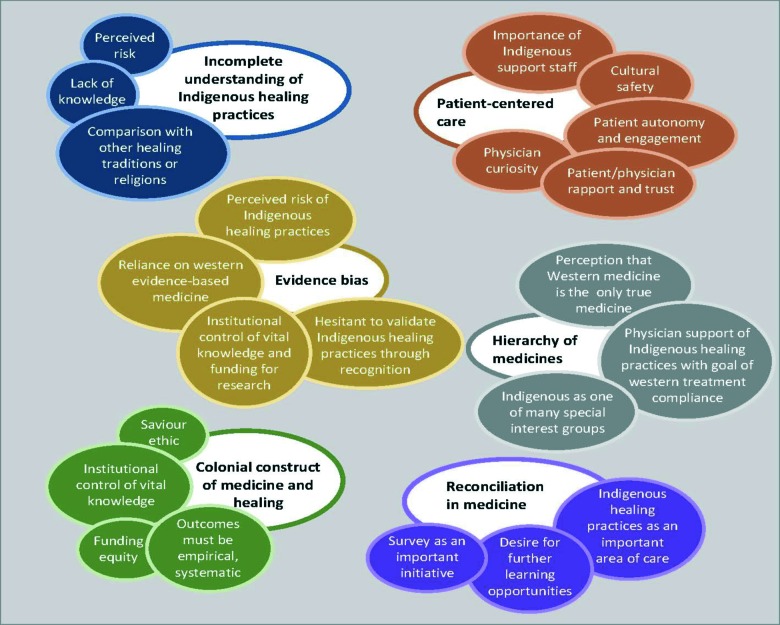
Overarching qualitative themes extracted from the text responses (larger, open bubbles) and their sub-themes (smaller, filled bubbles), with quotes from respondents that illustrate the themes.

Figure 2 summarizes the themes expressed within the survey’s free-text responses. The importance of **patient-centred care** stood out, as respondents recognized that building rapport and trust, and patients having “*agency in their care*” (respondent 73) leads to better outcomes. Respondents generally felt that “*asking about it [Indigenous healing practices] helps build trust*” (respondent 29). They also expressed the view that the patient-physician relationship could benefit from learning more about these practices and from a collaborative treatment approach, such as one that is “*team based with elders/healers*” (respondent 21).

The free-text responses also reflect an **incomplete understanding of Indigenous healing practices**. Many respondents stated that they need more information, and many described uncertainty about specific Indigenous healing practices (e.g., ingested/inhaled substances) and potential risks. There was also frequent conflation of Indigenous healing practices with forms of complementary medicine or religions. This was demonstrated with responses such as: “*religious beliefs need to be kept separate from medicine*” (respondent 6), and “*I ask about all therapies the patient is using and specify ‘natural, vitamins, herbal or other*’” (respondent 19).

We also identified **evidence bias** as a major theme.In line with the dominance of positivism in science and medicine,respondents indicated a desire for biomedical-type evidence on Indigenous healing practices (e.g., peer-reviewed, published, randomized-controlled trial data). This desire demonstrates a lack of understanding of Indigenous knowledge and approaches to evidence, including accumulation of empirical observations passed down through oral tradition. This was evident when a respondent noted that “*this is currently an evidence-free zone*” (respondent 62). This evidence bias seems to imply a **hierarchy of medicines**, in which biomedicine is believed to provide the best treatment, and other healing practices can be tolerated as long as they do not interfere with the effectiveness of or adherence to biomedical treatment plans. This emerged when one respondent argued that “*the acceptance of alternative therapeutic practices distracts individuals from appropriate therapy*” (respondent 48).

Overall, the free-text responses reflect a **colonial construct of medicine and healing**. While the research team agreed that most respondents truly seemed to care for their patients’ well-being, the physicians’ comments also reflected a “saviour ethos” around the place and purpose of western medicine, a theme that is central to colonization. This emerged in words like, “*there comes a time when it becomes heartbreaking to see how the Indigenous patient does not want Western Rx medicine even when it is the standard of care worldwide*” (respondent 7).

Many of the free-text responses recognize Indigenous healing practices as an important area of care “*chiefly hampered by lack of information*” (respondent 53), and most respondents expressed a desire for further learning opportunities. Survey responses show support for **reconciliation in medicine**, for healing relationships through constructive action and creating a more equitable and inclusive society. Many respondents expressed a desire to build stronger, trust-based relationships, however their vision of reconciliation is framed by colonial paradigms: “*We should be open to study any traditional practice requested and federal reconciliation programs should fund this. Indigenous healers should be actively included in this process. Helpful practices should be incorporated into common practice with appropriate endorsement and credit given*” (respondent 48). Reflected in respondents’ support for reconciliation is the need to better understand the reconciliation process.

## Discussion

This study was an exploration of rheumatologists’ perceptions of Indigenous healing practices and intended to elucidate barriers to including these in care planning, with our ultimate goal being to develop strategies to bridge divergent worldviews and uphold Indigenous rights. These actions are critical given the impact arthritis has on Indigenous populations’ wellbeing, in the setting of multiple physical, mental, spiritual and emotional traumas incurred from historical and ongoing acts of colonization. Overall, we interpret the support for Indigenous healing practices as cautious. Although the survey findings revealed an expressed willingness by most respondents to learn more about Indigenous healing practices, inquire about the use of these practices, and to include these practices in patient care plans, qualitative responses demonstrated hesitancy about the place of Indigenous healing practices in relation to western-based therapies, or how to approach learning.

Exposure to Indigenous populations, such as through residing in a province with a proportionally increased number of Indigenous people, or being engaged in clinical work in Indigenous communities, increased comfort for these aspects. Although rheumatologists appeared willing to recognize the importance of Indigenous health practices to their patients’ holistic wellbeing, there remained a strong sense that they would only do so if the practices were tested and shown to fulfill their western paradigm of understanding. This speaks to unawareness of existing data in support of healing practices, such as the positive impacts of the Aboriginal Healing Foundation programming,^16^ but also perhaps the dominance of reliance on randomized-controlled study data which has catapulted paradigm-changing treatment approaches in rheumatology practice in the past two decades. This is best reflected in the concerns expressed about the safety and efficacy of these practices, as respondents felt that these practices could supplement symptom management and preventive health maintenance, but expressed resistance to their role in disease activity suppression. This is not unlike the findings of Zubek’s 1994 survey of family physicians in British Columbia,^7^ on which our survey was based, in that rheumatologists were less willing to agree with the use of Indigenous healing practices for more severe or inpatient conditions. Further exploration and conceptualization of the role of healing practices in rheumatology in ways that respects both Indigenous and western-based knowledges is required.

Further, our analysis of the free-text responses suggests that standard physician goals in treating the biological aspects of the disease may not match the goals of Indigenous patients, whose distinct cultural approaches may uphold different sets of values and expectations of medicine and healing.^17^ This difference in goals between western practitioners and Indigenous patients has been extensively researched in Canada with regards to Indigenous peoples with diabetes,^3^^,^^18^ with current clinical practice guidelines upholding that it is fundamental that diabetes care for Indigenous people be delivered in a manner that includes traditional and cultural approaches to wellness.^4^^,^^18^^,^^19^ However, the qualitative results do suggest support for “patient-centered care,” whereby rheumatologists who engaged their patients in conversations about Indigenous healing practices felt that they provided those patients with more agency in their care. These findings parallel a recent study showing that support for and access to Indigenous healing practices in an urban setting increases empowerment and self-determination within healthcare relationships.^5^ Indeed, it is well known that patients with chronic conditions have many strategies to become invested and activated in their own care.^20^ The agency and understanding from this result in better health outcomes.^21^

The overwhelming majority of respondents agreed with the involvement of Indigenous healing practices in many levels of patient care, but a way forward to create space for these in patient care plans needs to be offered to pursue this approach to reconciliation in medicine. By large, respondents indicated a willingness to attend learning sessions at conferences, thus our group has offered a dedicated workshop at each annual scientific meeting of our professional organization. This session purposefully introduces two-eyed seeing to support learning and reflection of health practitioners to gain understanding of the values and knowledge generation of Indigenous healing practices from the lens of Indigenous peoples, rather than from the traditional framework of positivism and “western” scientific evidence.^22^At the broader system level, ongoing efforts to decolonize medical education at all levels of learning are to be pursued,^23^ as is change within the health system structures for care delivery. Concrete steps forward have recently been proposed in Canada through the approval of the Joint Commitment to Action on Indigenous Health by the Association of Faculties of Medicine of Canada Board of Directors,^24^ and the passing of a resolution to include Indigenous health education across all specialty residency training programs by the Royal College of Physicians and Surgeons of Canada.^25^ In the health system, individual practitioners and broader health organizations should continue to seek out suitable approaches and work in collaboration with Indigenous communities and leadership to enact this right within the health system.^26^ As proposed by Greenwood, change is required at structural (e.g. legislation, policy, agreements), systemic (eg organizations, programs, systems), and service delivery (cultural safety, individual) levels.^27^ At all times, Indigenous rights to health and regaining self-determination over all aspects of decision-making and service delivery are overarching principles. This is a solid structured approach by which to consider how providers can begin to imagine their place in supporting Indigenous community ownership and delivery of health services.

Limitations of our study include the 15% response rate, despite a reminder to complete the survey, and strong support of the CRA for Indigenous Health and Equity initiatives. Additional data collection techniques, such as interviews and focus groups, would expand understanding of physician awareness, acceptance and hesitancy for Indigenous healing practices in clinical care plans. We unintentionally omitted gender and sex as participant demographic characteristics in our data collection, and this would be another potential modifier for results, but with no existing data to know if physician gender impacts acceptance of Indigenous healing practices. Future work is directed to leaders of medical education institutions and those providing continuing professional development opportunities, as well as to health systems leadership who are urged to consider structural supports that promote safe care environments, as demonstrations of a commitment to reconciliation and the Indigenous population.

### Conclusion

The study results support that physicians appear ready to be engaged in reconciliation acts in medicine, with interest in learning about and understanding Indigenous healing practices, and including them in patient care plans. We anticipate a cautious approach will be taken as physicians wrestle with accepting knowledges and approaches to evidence-generation that are not familiar to them. If the medical profession truly intends to support patient-centered care, it is imperative that practitioners at the very least respect the importance of Indigenous knowledge and traditional healing approaches for their Indigenous patients. It is important for physicians not to judge, nor to simply learn about Indigenous healing practices, but rather to create space for these practices as an act of reconciliation. Broader application of the survey to assess physician readiness to the inclusion of Indigenous healing practices, such as in primary care, other specialties, and in both academic and non-academic settings would strengthen the conclusions and set direction for medical professionals and those who can effect change in health systems to inform culturally appropriate treatment guidelines, training programs, and ultimately the delivery of medical services to Indigenous patients.

## References

[1] Truth and Reconciliation Commission of Canada Honouring the truth, reconciling for the future. Summary of the final report of the Truth and Reconciliation Commission of Canada.; 2015.

[2] Government of Canada. Indian Act, RSC 1985, c I-5 Available from: https://laws-lois.justice.gc.ca/PDF/I-5.pdf. [Accessed February 25, 2020]

[3] Jacklin KM, Henderson RI, Green ME, Walker LM, Calam B, Crowshoe LJ Health care experiences of Indigenous people living with type 2 diabetes in Canada. Cmaj. 2017;189(3):E106-E12. 10.1503/cmaj.161098 28246155 PMC5250516

[4] Crowshoe LL, Henderson R, Jacklin K, Calam B, Walker L, Green ME Educating for equity care framework: Addressing social barriers of Indigenous patients with Type 2 diabetes. Can Fam Physician. 2019;65(1):25-33. 30674510 PMC6347314

[5] Auger M, Howell T, Gomes T Moving toward holistic wellness, empowerment and self-determination for Indigenous peoples in Canada: Can traditional Indigenous health care practices increase ownership over health and health care decisions? Canadian journal of public health = Revue canadienne de sante publique. 2016;107(4-5):e393-e8. 10.17269/CJPH.107.5366 28026704 PMC6972123

[6] Oster RT, Grier A, Lightning R, Mayan MJ, Toth EL Cultural continuity, traditional Indigenous language, and diabetes in Alberta First Nations: a mixed methods study. International journal for equity in health. 2014;13:92 10.1186/s12939-014-0092-4 25326227 PMC4210509

[7] Chandler MJ, Lalonde CE Cultural continuity as a hedge against suicide in Canada’s First Nations. Transcultural Psychiatry. 1998;35(2):191-219. 10.1177/136346159803500202

[8] Rogers BJ, Swift K, van der Woerd K, Auger M, Halseth R, Atkinson D, et al At the interface: Indigenous health practitioners and evidence-based practice. Prince George, BC: National Collaborating Centre for Aboriginal Health; 2019.

[9] World Health Organization. WHO Traditional Medicine Strategy 2014-2023. 2014.

[10] UN General Assembly United Nations declaration on the rights of Indigenous Peoples : resolution/adopted by the General Assembly.; 2007 2 10 2007. Contract No.: A/RES/61/295.

[11] Truth and Reconciliation Commission of Canada Truth and Reconciliation Commission of Canada: Calls to action. Winnipeg, MB; 2015.

[12] Barnabe C, Jones CA, Bernatsky S, Peschken CA, Voaklander D, Homik J, et al Inflammatory arthritis prevalence and health services use in the First Nations and Non-First Nations populations of Alberta, Canada. Arthritis Care Res (Hoboken). 2017;69(4):467-74. 10.1002/acr.22959 27333120

[13] Barnabe C, Crane L, White T, Hemmelgarn B, Kaplan GG, Martin L, et al Patient-reported outcomes, resource use, and social participation of patients with rheumatoid arthritis treated with biologics in Alberta: Experience of Indigenous and Non-indigenous patients. J Rheumatol. 2018;45(6):760-5. 10.3899/jrheum.170778 29449496

[14] Zubek EM Traditional Native healing. Alternative or adjunct to modern medicine? Can Fam Physician. 1994;40:1923-31. 7841824 PMC2380248

[15] Cantrill JA, Sibbald B, Buetow SA The Delphi and nominal group techniques in health services research. Int J Pharm Pract. 1996;4:67-74. 10.1111/j.2042-7174.1996.tb00844.x

[16] The Aboriginal Healing Foundation. Summary Points of the AHF Final Report. Ottawa, ON; 2006.

[17] Brant CC Native ethics and rules of behaviour. Can J Psychiatry. 1990;35(6):534-9. 10.1177/070674379003500612 2207989

[18] Diabetes Canada Clinical Practice Guidelines Expert C, Crowshoe L, Dannenbaum D, Green M, Henderson R, Hayward MN, et al Type 2 Diabetes and Indigenous Peoples. Can J Diabetes. 2018;42 Suppl 1:S296-S306. 10.1016/j.jcjd.2017.10.022 29650108

[19] Aspin C, Brown N, Jowsey T, Yen L, Leeder S Strategic approaches to enhanced health service delivery for Aboriginal and Torres Strait Islander people with chronic illness: a qualitative study. BMC Health Serv Res. 2012;12:143 10.1186/1472-6963-12-143 22682035 PMC3405462

[20] Jowsey T, Dennis S, Yen L, Mofizul Islam M, Parkinson A, Dawda P Time to manage: patient strategies for coping with an absence of care coordination and continuity. Sociol Health Illn. 2016;38(6):854-73. 10.1111/1467-9566.12404 26871716

[21] Greene J, Hibbard JH Why does patient activation matter? An examination of the relationships between patient activation and health-related outcomes. Journal of general internal medicine. 2012;27(5):520-6. 10.1007/s11606-011-1931-2 22127797 PMC3326094

[22] Martin DH Two-eyed seeing: a framework for understanding Indigenous and non-Indigenous approaches to Indigenous health research. Can J Nurs Res. 2012;44(2):20-42. 22894005

[23] Jones R, Crowshoe L, Reid P, Calam B, Curtis E, Green M, et al Educating for Indigenous health equity: an International consensus statement. Acad Med. 2019;94(4):512-9. 10.1097/ACM.0000000000002476 30277958 PMC6445615

[24] Writing working group on behalf of the Indigenous health network joint commitment to action on Indigenous health. Ottawa, ON: Association of Faculties of Medicine of Canada; 2019.

[25] Indigenous health is a priority for the Royal College [press release]. Ottawa, ON2017.

[26] Park YL, Canaway R Integrating traditional and complementary medicine with national healthcare systems for universal health coverage in Asia and the Western Pacific. Health Syst Reform. 2019;5(1):24-31. 10.1080/23288604.2018.1539058 30924749

[27] Greenwood M Modelling change and cultural safety: A case study in northern British Columbia health system transformation. Healthcare Manage Forum. 2019;32:11-4. 10.1177/0840470418807948 30514119

